# The Effect of *Turnera diffusa* Leaf Supplementation in Diet on the Qualitative and Quantitative Characteristics of Boar Semen

**DOI:** 10.3390/life16010083

**Published:** 2026-01-06

**Authors:** Mariyana Petrova, Gergana Yordanova, Katya Eneva, Radka Nedeva, Krum Nedelkov, Toncho Penev

**Affiliations:** 1 Agricultural Institute, 9700 Shumen, Bulgaria; mari_anna1305@abv.bg (M.P.); gerganaarshspb@abv.bg (G.Y.); katiqeneva@abv.bg (K.E.); r.nedeva@abv.bg (R.N.); 2 Agricultural Academy, 1373 Sofia, Bulgaria; 3 Department of General Animal Husbandry, Trakia University, 6000 Stara Zagora, Bulgaria; krum.nedelkov@trakia-uni.bg; 4 Department of Ecology and Animal Hygiene, Trakia University, 6000 Stara Zagora, Bulgaria

**Keywords:** *Turnera diffusa* leaves, semen quality, boars

## Abstract

The present study aimed to investigate the effect of *Turnera diffusa* supplementation on the quantitative and qualitative characteristics of semen in Duroc boars (*n* = 4). The experiment was divided into two periods, each corresponding to the duration of one spermatogenic cycle: a control period (40 days) (CP) and an experimental period (40 days) (EP). Nutrition and environmental conditions were kept constant throughout both periods. During the experimental period, each boar received a daily supplement of 7 g of *Turnera diffusa* extract. In each period, five ejaculates were collected from each boar included in the study. The ejaculates were evaluated for volume, sperm concentration, motility, agglutination, number of insemination doses obtained per ejaculate after dilution, and sperm viability after 24, 48, and 72 h of storage. The results of a two-way repeated measures ANOVA showed that the combined effect of boar × treatment significantly influenced ejaculate volume (*p* < 0.01) and viability after 48 h of storage (*p* < 0.05). The results of the two-way repeated measures ANOVA showed that treatment with the tested additive *T. diffusa* significantly affected sperm survival during storage for 24 h (*p* < 0.01), 48 h (*p* < 0.001), and 72 h (*p* < 0.05). Bonferroni post hoc analysis indicated that *T. diffusa* significantly affected only the parameters related to sperm viability, namely survival rates at 24 h (*p* < 0.001), 48 h (*p* < 0.01), and 72 h (*p* < 0.01). The findings of this study demonstrate that the application of the tested supplement, at the specified dose and duration, has a positive effect on semen quality in boars.

## 1. Introduction

Fertility in female pigs has a more pronounced influence on reproductive efficiency in swine production compared to the fertility of boars [1]. However, a considerable replacement rate is also observed among boars, primarily due to the deterioration of semen quality [2]. Artificial insemination is a fundamental biotechnological method in pig reproduction [3], and its effectiveness depends largely on semen quality [4,5]. In recent years, alongside conventional additives [6,7,8], the swine industry has increasingly sought natural plant-based alternatives to enhance the reproductive performance of boars. Various plant extracts are used for this purpose, aiming to improve reproductive capacity and sperm production in animals [9,10].

*Turnera diffusa*, a plant commonly known as Damiana, has an ancient origin. Studies evaluating the effects of *T. diffusa* on testicular function under conditions of toxic exposure have demonstrated beneficial outcomes, including reduced lipid peroxidation, enhanced antioxidant activity, and improved hormonal status in treated animals [11,12]. Some studies indicate that *T. diffusa* extract exerts a positive influence on male reproductive function and semen quality. Parra-Naranjo et al. [13] describe the bioactive properties of plants from the *Turnera* genus. Experimental models show that the plant reduces the latency to ejaculation and shortens the post-ejaculatory interval in male rats, which is associated with increased sexual activity and improved androgenic functional balance [14]. The main active compounds—flavonoids (including apigenin and its glycosides)—exhibit vasodilatory effects on the smooth muscle of the penile corpus cavernosum through activation of nitric oxide (NO) and cyclic guanosine monophosphate (cGMP) signaling pathways [14].

Furthermore, *T. diffusa* extract has demonstrated protective effects on the testes against toxic damage induced by pesticides (such as fenitrothion) [11] and drugs such as amitriptyline [12]. These effects are attributed to the plant’s pronounced antioxidant potential, which neutralizes reactive oxygen species (ROS) and protects sperm cell membranes and DNA from oxidative stress.

Estrada-Reyes et al. [15] reported that in an experiment with mice exhibiting induced obesity and impotence, oral administration of an aqueous extract of *T. diffusa* led to increased libido and restoration of copulatory ability. According to Chevallier [16], the α-sitosterol contained in the plant may exert an effect similar to that of testosterone, stimulating hormonal secretion, influencing metabolism, and supporting growth, reproductive processes, sexual behavior, and the development of the male gonads [17,18]. Oral administration of 50 mg/kg *T. diffusa* extract to rats exposed to fenitrothion and potassium dichromate resulted in the restoration of testicular structure and an increased sperm count, indicating a protective effect of *T. diffusa* against induced testicular toxicity [11,19].

Palacios et al. [20] reported that the injection of lyophilized *T. diffusa* in 60-day-old male piglets at a dose of 20 mg/2 mL distilled water resulted in a statistically significant increase in testicular circumference and weight, as well as epididymal length. Another positive effect reported by the authors is the increase in ejaculate volume, sperm concentration, and sperm motility in the treated pigs.

*Turnera diffusa* extract has been supplemented among laboratory animals, primarily rats, as well as in humans, focusing on its aphrodisiac, antioxidant, and hormone-regulating properties. To date, research specifically examining the effects of *T. diffusa* leaf on reproductive function in swine is limited. This observation motivated our initiative to conduct further experiments aimed at determining the potential impact of this bioactive supplement on both the qualitative and quantitative characteristics of boar semen. On the Bulgarian market, *T. diffusa* is available in the form of dried leaf material, capsules, and tincture.

The aim of the present study was to determine the effect of including leaf (*Turnera diffusa*) in the diets for boars on semen quantity and quality, assessed through a number of reproductive parameters.

## 2. Materials and Methods

### 2.1. Animals, Experimental Design, and Feeding

This study was conducted at “Farma Kapitanova” Ltd., in the town of Ignatievo, Varna. Four boars were randomly assigned to treatments as a sample from a population of a synthetic (terminal) Duroc line, with an average live weight of 288.5 ± 14 kg and an average age of 18.75 ± 1.75 months, over a period of 80 days. All boars included in the study exhibited excellent reproductive health status. The experiment was divided into two sub-periods, each corresponding to the duration of a single spermatogenic cycle: a control period (40 days), referred to in the study as CP, and an experimental period (40 days), referred to as EP. During both sub-periods, the boars were fed a compound feed with a defined ingredient composition and nutrient profile adjusted to their physiological requirements presented in Table 1.

Feeds were analyzed using wet chemistry methods for dry matter, crude protein, crude fiber, ether extract, calcium and phosphorus as described by AOAC International (2007) [21]. Amino acid profiles were analyzed by using the HPLC/ULPC (High-Performance Liquid Chromatography/Ultra-Performance Liquid Chromatography) method with derivatization.

During the experimental period, each boar received a daily hand-administered supplement of 7 g/head/day of dried *T. diffusa* leaf material. These dried leaves were purchased from a human dietary supplement drugstore, origin from Mexico. The supplemented amount was based on the dosage reported by Estrada-Reyes et al. [14], corresponding to 25 mg/kg body weight. Since, in the present study, two of the boars weighed just over 300 kg, one weighed 296 kg, and only one weighed 280 kg, a uniform dosing approach was adopted, whereby all boars received the same daily supplementation of 7 g. Its chemical composition includes triacontane, β-sitosterol, hexaconazole, and 5-hydroxy-7,3,4-trimethoxyflavone (0.4–0.5%) [22], as well as α- and β-pinene, p-cymene, and 1,8-cineole (0.5–0.9%), tannins (4%), resins (7%), and cyanogenic glycosides. The daily amount of compound feed per boar was (Boar No. 1—3.1 kg, No. 2—3.3 kg, No. 3—3.0 kg, No. 4—3.2 kg), with an average of 3.15 ± 0.1 kg, adjusted to the age (Boar No. 1—20 months; No. 2—18 months; No. 3—21 months; No. 4—16 months) and live weight (Boar No. 1—305 kg; No. 2—300 kg; No. 3—296 kg; No. 4—280 kg) of the animals. The addition of *T. diffusa* leaf to the ration was carried out in the morning between 8:00 and 9:00 a.m. The microclimate in the boar housing facility remained unchanged during both periods and was regulated by an automated electronic control system manufactured by BIG DUTCHMAN (Vechta, Lower Saxony, Germany).

### 2.2. Semen Collection and Quantitative and Qualitative Analyses

To ensure comparable workload during the control period (CP) and the experimental period (EP), five ejaculates were collected from each boar in both periods at 7-day intervals, resulting in a total of 40 ejaculates. Semen collection was performed in a designated collection room using the manual “gloved-hand” technique according to Admal [23]. The ejaculate was collected into disposable collection bags fitted with filter paper. After removal of the mucinous fraction, the filtered semen was transferred into a sterile beaker pre-warmed to 37 °C and graduated for the determination of ejaculate volume. An organoleptic assessment was performed, including evaluation of color, density, and odor. The assessment of sperm viability at 24, 48 and 72 h was carried out via microscopic assessment at the respective hour. After this the parameters sperm motility and agglutination were examined also microscopically. Sperm concentration was determined using a sperm counter (Ibersan—Materials and Equipment for Artificial Insemination, Santiago do Cacém, Portugal). Sperm concentration was evaluated before dilution. Based on the reading obtained from the device, the corresponding sperm concentration (million/mL) was calculated using the conversion table provided with the equipment (Supplementary Table S1). The ejaculate was diluted using the semen extender MS Dilufert—Gold (manufacturer: MS Schippers—Hapert, Noord Brabant, The Netherlands) at a 1:1 ratio. Sperm motility and agglutination were evaluated under a PRO-Biolar 12036 microscope at 10×/25× magnification (Warsaw, Poland). If no changes were observed in the already 1:1 diluted semen, complete dilution of the ejaculate was performed to prepare insemination doses, using the data on volume, density, and motility. A final microscopic assessment was conducted on the semen ready for insemination. The diluted semen was stored in a dedicated refrigerated cabinet (Liebherr, Germany) at 15–16 °C. Prior to use, the semen underwent another microscopic evaluation to determine sperm motility and the presence of agglutination.

During the experiment, the following parameters were monitored:

- Ejaculate volume (mL);
- Sperm concentration (million/mL);
- Sperm motility (%);
- Sperm agglutination (%);
- Number of insemination doses obtained from one ejaculate;
- Sperm viability at 24, 48, and 72 h.

The number of insemination doses obtained from a single ejaculate was calculated using a formula ensuring 3.5 billion motile sperm cells per dose, as proposed by Trevor Evans (Pig International, 1994) [24]:

N = (d × v)/D × m/10 × c/100;

- N—number of doses;
- D—number of sperm cells required per dose;
- d—sperm concentration (million/mL);
- v—ejaculate volume (mL);
- m—motility coefficient expressed as a fraction (e.g., 70% motility = 0.7);
- c—coefficient representing the percentage of morphologically normal sperm, expressed analogously to the motility coefficient.

### 2.3. Statistical Analysis

The normality of data distribution for each semen parameter was assessed using the Shapiro–Wilk test. For parametric data, a T-test was applied, and for nonparametric data, the Wilcoxon test was applied. To evaluate the effects of the studied factors—*T. diffusa* leaf supplementation and boar identity—as well as their combined interaction (boar × treatment) on the semen parameters, a two-way repeated measures ANOVA followed by the Bonferroni post hoc test. Differences were considered significant at *p* < 0.05. was performed. Statistical analyses were conducted using GraphPad Prism, version 10.6.1. (GraphPad Software, San Diego, CA, USA).

## 3. Results

Table 2 presents the results of the Shapiro–Wilk test assessing the normality of data distribution for the studied semen parameters in boars. The analysis showed that only ejaculate volume and the number of doses per ejaculate followed a normal distribution, whereas the remaining parameters did not meet the criteria for normality.

**Figure 1:**
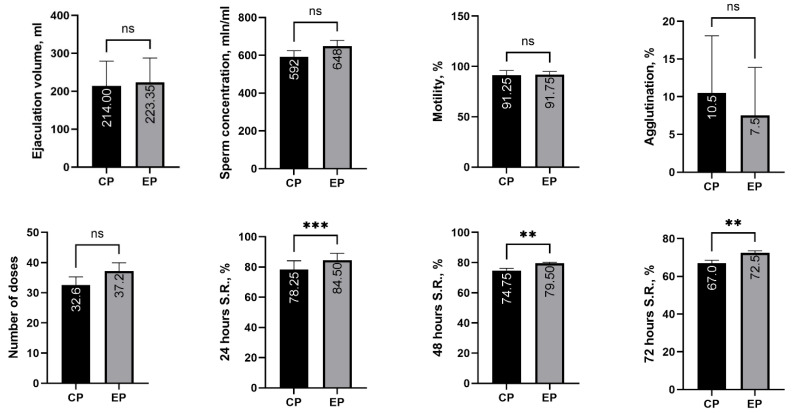
Comparative analysis of the investigated parameters across the study period. Note: ** *p* < 0.01; *** *p* < 0.001, n.s—non significance; CP—Control Period; EP—Experimental Period; S.R.—Survaival Rate.

Figure 1 presents a comparative analysis of the investigated semen parameters in boars (*n* = 4) during the control period (CP) and the experimental period (EP), along with the corresponding significance levels.

In the present study, ejaculate volume and sperm concentration increased during the experimental period by 4.21% and 9.46%, respectively, and these changes may have potential biological significance. Regarding the effect of the tested supplement on sperm motility, the difference was minimal, with only a 0.55% increase in the experimental period compared to the control; however, this change may still indicate a potential biological benefit. For sperm agglutination, the difference amounted to 28.57%, with lower values observed during the experimental period. Agglutination represents the adhesion of spermatozoa to one another at various structural points, which subsequently reduces their fertilizing capacity. Therefore, although the lower agglutination rate observed in the experimental period was not statistically significant, it still indicates a potentially beneficial effect on sperm fertilizing ability.

For the parameter “number of insemination doses per ejaculate,” 4.60 more doses were obtained during the experimental period compared to the control, representing an increase of 14.1%. For sperm viability at 24 h, the difference between periods was 6.25 percentage points, representing an approximately 8% increase during the experimental period, with a significance level of *p* < 0.001. A similar trend was observed for viability at 48 h, where the value was 4.75 percentage points higher an increase of 6.35%—in the experimental period (*p* < 0.01). For 72 h sperm viability, the difference between the control and experimental periods was 8.21%, with a significance level of *p* < 0.01. To determine the influence of the *T. diffusa* supplement on the studied semen parameters, as well as the effect of the factor “Boar”, a factorial two-way repeated measures ANOVA was performed. The results of this analysis are presented in Table 3. The findings indicate that the “Boar” factor significantly affected ejaculate volume, sperm concentration, number of doses per ejaculate, and sperm viability at 24, and 72 h, all with a high level of significance (*p* < 0.001). Assessment of the combined Boar × Treatment interaction showed significant effects on ejaculate volume (*p* < 0.01) and viability at 48 h (*p* < 0.05). With respect to the treatment factor alone, significant effects were observed for sperm viability at 24 h (*p* < 0.01) and 48 h (*p* < 0.001), as well as for 72 h viability (*p* < 0.05).

## 4. Discussion

In the present study, increases in ejaculate volume, sperm concentration, the number of insemination doses per ejaculate, and sperm viability at 24, 48, and 72 h were observed during the experimental period compared with the control (Figure 1). These results may be attributed to several biological effects associated with *T. diffusa* leaves. Sperm motility, indicating the ability of spermatozoa to move actively, and sperm agglutination, representing the tendency of spermatozoa to cluster together, were assessed as key indicators of semen quality and fertilizing potential in boars. High motility and low agglutination are associated with improved reproductive performance. Previous studies have shown that this supplement enhances testosterone, follicle-stimulating hormone (FSH), luteinizing hormone, and prolactin levels after four weeks of administration [12]. In another four-week study, significant improvements in the Johnsen score, the blood–testis protein barrier, sperm motility, viability, and sperm count were reported in treated animals [19]. These findings support the results of the current study, in which increases in ejaculate volume and a reduction in sperm agglutination were also observed. Other studies have similarly demonstrated the protective effects of *T. diffusa* against oxidative stress and various testicular insults [12,13]. The positive influence of *T. diffusa* is often attributed to its protective and supportive role on Sertoli cells, which are essential for the development and maturation of spermatozoa [19].

In the current study, sperm agglutination decreased by 28.57% during the experimental period, while the number of doses per ejaculate increased by 14.1%. Similar findings were reported by Palacios et al. [20], who demonstrated that treatment with *T. diffusa* improved sperm morphological abnormalities in boars by approximately 14%. This is consistent with—and helps to explain—the lower agglutination values observed in our study. It should be noted, however, that the trial conducted by Palacios et al. [20] lasted only 21 days, whereas the present experiment was carried out over 40 days. This indicates that further research is needed to clarify the effects of dosage and treatment duration when using *T. diffusa* as a biostimulant for improving boar semen quality. Importantly, statistically significant differences between the control and experimental periods were found for sperm viability at 24, 48, and 72 h.

During storage, boar sperm typically undergo several detrimental changes, including reductions in motility, viability, and membrane integrity, due to their high sensitivity to peroxidative damage [25]. According to the authors, these changes result from the relatively high proportion of polyunsaturated fatty acids in the phospholipid membrane of boar spermatozoa. Another contributing factor to reduced sperm survival during storage is the comparatively low antioxidant activity of boar seminal plasma [26]. These negative changes, however, were not observed in the present study. Instead, the data demonstrate that *T. diffusa* supplementation markedly improved sperm viability at 24, 48, and 72 h of storage.

This outcome is supported by the well-documented pharmacological properties of *T. diffusa*, one of which is its ability to enhance antioxidant activity [13]. Furthermore, the supplement demonstrates oxygen radical absorbance capacity (ORAC), allowing it to neutralize free radicals effectively [27]. Together, these mechanisms explain the significant improvements in sperm viability during storage observed in the current study. Table 3 presents the results of the two-way repeated measures ANOVA assessing the effects of the factors boar, treatment, and their interaction (boar × treatment) on the examined semen parameters. The data show that the boar factor exerted a significant influence on nearly all evaluated parameters, with the exception of agglutination. These traits are directly associated with the ability of sperm cells to reach the oocyte and achieve fertilization. As sperm motility increases, the farrowing rate—expressed as the percentage of sows that successfully farrow—also increases [28]. Sperm motility is influenced by the intracellular production of cyclic adenosine monophosphate (cAMP) [29]. To date, no studies have demonstrated a direct effect of *T. diffusa* supplementation on cAMP levels. Whether *T. diffusa* supplementation directly influences intracellular cAMP production will be addressed in future research. However, some natural supplements have been shown to affect mechanisms such as antioxidant activity and hormone regulation, thereby exerting a stimulatory effect on spermatozoa [30]. It is plausible that *T. diffusa* may have a similar stimulatory influence on motility, although further research is needed to confirm this hypothesis.

The ANOVA results also revealed that the combined boar × treatment interaction significantly affected ejaculate volume (*p* < 0.01) and sperm viability at 48 h (*p* < 0.05). Ejaculate volume is influenced by factors related to the individual developmental trajectory of each boar [31], which are unlikely to be modified by the tested supplement. Ambient temperature is another key determinant of ejaculate volume. In the present study, all boars were kept under identical climatic conditions during both the control and experimental periods. Therefore, it can be concluded that ejaculate volume was determined primarily by the boar factor and was not related to *T. diffusa* supplementation.

The results of the two-way repeated measures ANOVA (Table 3) showed that treatment with the tested additive *T. diffusa* significantly affected sperm survival during storage at 24 h (*p* < 0.01), 48 h (*p* < 0.001), and 72 h (*p* < 0.05).

The results of the Bonferroni post hoc test for the effect of the tested additive are presented in Figure 1. The data show that *T. diffusa* significantly affected only the parameters related to sperm viability. Specifically, sperm viability was significantly higher in the *T. diffusa*–treated group at 24 h (*p* < 0.001), 48 h (*p* < 0.01), and 72 h (*p* < 0.01) (Figure 1). These parameters are known to be affected by factors such as semen collection frequency, bacterial or viral contamination, and semen handling procedures following collection. In the present study, all such factors were carefully controlled through the selection of animals, the structure of the study periods, and the environmental conditions. Therefore, the observed improvements in sperm viability are attributed to the dietary supplementation with *T. diffusa*. It has been demonstrated that boar semen quality can be modulated through nutritional strategies aimed at reducing oxidative stress, thereby extending sperm storage longevity [32], which aligns with the findings of the present study.

Supplementation with *T. diffusa* has been shown to improve lipid peroxidation, enzymatic activity, and hormonal status, all of which contribute to enhanced semen quality and increased sperm survival [11]. These mechanisms are consistent with the significant improvements in viability observed in this study.

## 5. Limitations

The relatively small number of animals (four boars) is considered a limitation of the present study. This limitation arises from the fact that the study was conducted on a private commercial farm, where the demand for boars of the same breed, age, and body weight is limited. Another limitation is the use of a uniform dosage of the tested supplement for all boars. This approach was adopted because the supplement was administered manually by animal caretakers. To minimize the risk of dosing errors across different work shifts, a standardized dosage of 7 g per animal per day was applied to all boars.

## 6. Conclusions

The present study demonstrated that supplementation with *T. diffusa* (Damiana) for a period of 40 days exerts a positive effect on semen quality in Duroc boars. The most pronounced benefit was the significant improvement in sperm viability during storage at 24, 48, and 72 h. Although no statistically significant differences were observed for some of the remaining semen parameters, clear positive trends were evident across all evaluated traits. These findings suggest that *T. diffusa* may have broader biological effects on boar reproductive performance. Further research is warranted to elucidate the underlying biological mechanisms through which *T. diffusa* influences semen characteristics in boars.

## Figures and Tables

**Table 1:** Ingredient and chemical composition of the diet fed in the experiment.

Item	Diet
Ingredient, % of diet	
Corn	21.0
Wheat	31.0
Barley	30.0
Sunflower meal, 34%	12.0
Wheat bran	4.0
Mineral–vitamin premix ^1^	2.0
Composition, % of DM unless otherwise noted	
DM, %	88.3
MEs, Mcal/kg ^2^	3.07
NEs, Mcal/kg ^2^	2.37
CP	17.0
CF	3.63
Crude fiber	5.11
Lysine	0.78
Methionine + Cystine	0.73
Threonine	0.71
Tryptophan	0.22
Calcium	0.84
Phosphorus, total	0.76
Digestible phosphorus	0.53
Vit A, IU/kg	12,000
Vit D3, kg	2000
Vit E, mg/kg	100

DM—dry matter; ME—metabolizable energy; NE—net energy; ^1^ The mineral–vitamin premix (Bulsou gesto 2%) contained (Crude protein (CP)—10.6%; Crude fat (CF)—0.60%; Crude fiber—1.10%; Crude ash—68.70%; Calcium—14.70%; Phosphorus—4.46%; Sodium—8.33%; Lysine—6.54%; Methionine—0.01%; Vitamin A—10.000 IU (from 500.000 IU); Vitamin D3—2.000 IU (from 100.000 IU); Vitamin E—50 mg/kg (from 2.500 mg/kg); Iron—6.000 mg/kg; Copper—600 mg/kg; Zinc—4.000 mg/kg; Manganese—2.000 mg/kg; Iodine—100 mg/kg; Selenium—15 mg/kg). ^2^ Values calculated using the chemical analysis of individual feed ingredients of the diet.

**Table 2:** Shapiro–Wilk test for normality of data distribution.

Indicator	Period	W Value	*p* Value	Normality
Ejaculation volume	Control	0.9275	0.138	Yes
	Experimental	0.9460	0.311	Yes
Sperm concentration	Control	0.8866	0.023 *	No
	Experimental	0.8898	0.027 *	No
Motility, %	Control	0.7176	<0.001 ***	No
	Experimental	0.7964	<0.001 ***	No
Agglutination, %	Control	0.8159	0.002 **	No
	Experimental	0.7795	<0.001 ***	No
Number of doses	Control	0.9735	0.826	Yes
	Experimental	0.9600	0.543	Yes
24 h S.R., %	Control	0.7546	<0.001 ***	No
	Experimental	0.8363	0.003 **	No
48 h S.R., %	Control	0.8004	<0.001 ***	No
	Experimental	0.6238	<0.001 ***	No
72 h S.R. %	Control	0.8151	0.001 **	No
	Experimental	0.7052	<0.001 ***	No

Significance: * *p* < 0.05; ** *p* < 0.01; *** *p* < 0.001.

**Table 3:** Two-way repeated measures ANOVA showing the effects of boar, treatment, and their interaction on semen parameters in boars (*n* = 4).

	Indicators				
	Ejaculation Volume				
Source	SS (Sum of Squares)	df (Degrees of Freedom)	MS (Mean Square)	F	*p*-Value
Boar	111,761	3	37,254	58.87	*p* < 0.001
Treatment	874.2	1	874.2	0.5564	*p* = 0.497
Boar × Treatment	17,265	3	5755	6.487	*p* = 0.007
Subject × Boar	7594	12	632.8		
Subject × Treatment	6285	4	1571		
Subject	6806	4	1701		
Residual	10,647	12	887.2		
	Sperm concentration				
Boar	512,640	3	170,880	30.08	*p* < 0.001
Treatment	31,360	1	31,360	3.087	*p* = 0.154
Boar × Treatment	5760	3	1920	0.3038	*p* = 0.822
Subject × Boar	68,160	12	5680		
Subject × Treatment	40,640	4	10,160		
Subject	56,000	4	14,000		
Residual	75,840	12	6320		
	Motility, %				
Boar	95.00	3	31.67	7.795	*p* = 0.004
Treatment	2.500	1	2.500	0.06015	*p* = 0.818
Boar × Treatment	72.50	3	24.17	2.667	*p* = 0.095
Subject × Boar	48.75	12	4.063		
Subject × Treatment	166.3	4	41.56		
Subject	166.3	4	41.56		
Residual	108.8	12	9.062		
	Agglutination, %				
Boar	20.00	3	6.667	0.1758	*p* = 0.911
Treatment	90.00	1	90.00	0.6729	*p* = 0.458
Boar × Treatment	90.00	3	30.00	0.6154	*p* = 0.618
Subject × Boar	455.0	12	37.92		
Subject × Treatment	535.0	4	133.8		
Subject	185.0	4	46.25		
Residual	585.0	12	48.75		
	Number of doses				
Boar	3447	3	1149	48.63	*p* < 0.001
Treatment	211.6	1	211.6	1.773	*p* = 0.254
Boar × Treatment	230.6	3	76.87	1.434	*p* = 0.282
Subject × Boar	283.5	12	23.63		
Subject × Treatment	477.4	4	119.4		
Subject	458.1	4	114.5		
Residual	643.4	12	53,62		
	24 h S.R., %				
Boar	456.9	3	152.3	18,99	*p* = 0.001
Treatment	390.6	1	390.6	27,78	*p* = 0.006
Boar × Treatment	61.88	3	20.63	0.9754	*p* = 0.411
Subject × Boar	96.25	12	8.021		
Subject × Treatment	56.25	4	14.06		
Subject	133.8	4	33.44		
Residual	253.8	12	21.15		
	48 h S.R., %				
Boar	396.9	3	132.3	13.37	*p* = 0.003
Treatment	225.6	1	225.6	103.1	*p* < 0.001
Boar × Treatment	241.9	3	80.63	7.101	*p* = 0.021
Subject × Boar	118.8	12	9.896		
Subject × Treatment	8.750	4	2.188		
Subject	66.25	4	16.56		
Residual	136.3	12	11.35		
	72 h S.R., %				
Boar	547.5	3	182.5	21.37	*p* < 0.001
Treatment	302.5	1	302.5	9.878	*p* = 0.035
Boar × Treatment	187.5	3	62.50	2.609	*p* = 0.137
Subject × Boar	102.5	12	8.542		
Subject × Treatment	122.5	4	30.63		
Subject	147.5	4	36.88		
Residual	287.5	12	23.96		

## Data Availability

The original contributions presented in this study are included in the article.

## Supplementary Materials

The following supporting information can be downloaded at: https://www.mdpi.com/article/10.3390/life16010083/s1, Table S1: Conversion Table (million/mL).
