# Supplementary material for: The Effect of Turnera diffusa Leaf Supplementation in Diet on the Qualitative and Quantitative Characteristics of Boar Semen
Source: Life (Basel). 2026 Jan 6;16(1):83. doi: 10.3390/life16010083 (PMC12843111; doi:10.3390/life16010083)
Supplement: Supplementary file 1 [file life-16-00083-s001.zip › life-4056521-supplementary.pdf]

Table S1. Conversion Table (million/ml)

Reading on device	Million/ml	Volume (in ml)																				
	Conc.	100.00	120.00	140.00	160.00	180.00	200.00	220.00	240.00	260.00	280.00	300.00	320.00	340.00	360.00	380.00	400.00	420.00	440.00	460.00	480.00	500.00
2.370	2.000	200.00	240.00	280.00	320.00	360.00	400.00	440.00	480.00	520.00	560.00	600.00	640.00	680.00	720.00	760.00	800.00	840.00	880.00	920.00	960.00	1000.00
2.350	1.920	192.00	230.00	268.80	307.20	345.60	384.00	422.40	460.80	499.20	537.60	576.00	614.40	652.80	691.20	729.60	768.00	806.40	844.80	883.20	921.60	960.00
2.340	1.840	184.00	220.00	257.60	294.40	331.20	368.00	404.80	441.60	478.40	515.20	552.00	588.80	625.60	662.40	699.20	736.00	772.80	809.60	846.40	883.20	920.00
2.310	1.760	176.00	211.20	246.40	281.60	316.80	352.00	387.20	422.40	457.60	492.80	528.00	563.20	598.40	633.60	668.80	704.00	739.20	774.40	809.60	844.80	880.00
2.290	1.680	168.00	201.60	235.20	268.80	302.40	336.00	369.60	403.20	436.80	470.40	504.00	537.60	571.20	604.80	638.40	672.00	705.60	739.20	772.80	806.40	840.00
2.160	1.600	160.00	192.00	224.00	256.00	288.00	320.00	352.00	384.00	416.00	448.00	480.00	512.00	544.00	576.00	608.00	640.00	672.00	704.00	736.00	768.00	800.00
2.120	1.520	152.00	182.40	212.80	243.20	273.60	304.00	334.40	364.80	395.20	425.60	456.00	486.40	516.80	547.20	577.60	608.00	638.40	668.80	699.20	729.60	760.00
1.080	1.440	144.00	172.80	201.60	230.40	259.20	288.00	316.80	345.60	374.40	403.20	432.00	460.80	489.60	518.40	547.20	576.00	604.80	633.60	662.40	691.20	720.00
1.040	1.360	136.00	163.20	190.40	217.60	244.80	272.00	299.20	326.40	353.60	380.80	408.00	435.20	462.40	489.60	516.80	544.00	571.20	598.40	625.60	652.80	680.00
1.010	1.280	128.00	153.60	179.20	204.80	230.40	256.00	281.60	307.20	332.80	358.40	384.00	409.60	435.20	460.80	486.40	512.00	537.60	563.20	588.80	614.40	640.00
1.950	1.200	120.00	144.00	168.00	192.00	216.00	240.00	264.00	288.00	312.00	336.00	360.00	384.00	408.00	432.00	456.00	480.00	504.00	528.00	552.00	576.00	600.00
1.883	1.120	112.00	134.40	156.80	179.20	201.60	224.00	246.40	268.80	291.20	313.60	336.00	358.40	380.80	403.20	425.60	448.00	470.40	492.80	515.20	537.60	560.00
1.810	1.040	104.00	124.80	145.60	166.40	187.20	208.00	228.80	249.60	270.40	291.20	312.00	332.80	353.60	374.40	395.20	416.00	436.80	457.60	478.40	499.20	520.00
1.750	0.960	96.00	115.20	134.40	153.60	172.80	192.00	211.20	230.40	249.60	268.80	288.00	307.20	326.40	345.60	364.80	384.00	403.20	422.40	441.60	460.80	480.00
1.660	0.880	88.00	105.60	123.20	140.80	158.40	176.00	193.60	211.20	228.80	246.40	268.00	281.60	299.20	316.80	334.40	352.00	369.60	387.20	404.80	422.40	440.00
1.610	0.800	80.00	96.00	112.00	128.00	144.00	160.00	176.00	192.00	208.00	224.00	240.00	256.00	272.00	288.00	304.00	320.00	336.00	352.00	368.00	384.00	400.00
1.470	0.720	72.00	86.40	100.80	115.20	129.60	144.00	158.40	172.80	187.20	201.60	216.00	230.40	244.80	259.20	273.60	288.00	302.40	316.80	331.20	345.60	360.00
1.370	0.640	64.00	76.80	89.60	102.40	115.20	128.00	140.80	153.60	166.40	179.20	192.00	204.80	217.60	230.40	243.20	256.00	268.80	281.60	294.40	307.20	320.00
1.240	0.560	56.00	67.20	78.40	89.60	100.80	112.00	123.20	134.40	145.60	156.80	168.00	179.20	190.40	201.60	212.80	224.00	235.20	246.40	257.60	268.80	280.00
1.080	0.480	48.00	57.60	67.20	76.80	86.40	96.00	105.60	115.20	124.80	134.40	144.00	153.60	163.20	172.80	182.40	192.00	201.60	211.20	220.80	230.40	240.00
0.930	0.400	40.00	48.00	56.00	64.00	72.00	80.00	88.00	96.00	104.00	112.00	120.00	128.00	136.00	144.00	152.00	160.00	168.00	176.00	184.00	192.00	200.00
0.780	0.320	32.00	38.40	44.80	51.20	57.60	64.00	70.40	76.80	83.20	89.60	96.00	102.40	108.80	115.20	121.60	128.00	134.40	140.80	147.20	153.60	160.00
0.650	0.240	24.00	28.80	33.60	38.40	43.20	48.00	52.80	57.60	62.40	67.20	72.00	76.80	81.60	86.40	91.20	96.00	100.80	105.60	110.40	115.20	120.00
0.440	0.160	16.00	19.20	22.40	25.60	28.80	32.00	35.20	38.40	41.60	44.80	48.00	51.20	54.40	57.60	60.80	64.00	67.20	70.40	73.60	76.80	80.00
0.210	0.080	8.00	9.60	12.20	12.80	14.40	16.00	17.60	19.20	20.80	22.40	24.00	25.60	27.20	28.80	30.40	32.00	33.60	35.20	36.80	38.40	40.00
